# Test your knowledge and understanding

**Published:** 2018-06-03

**This page is designed to help you to test your own understanding of the concepts covered in this issue, and to reflect on what you have learnt.**

**Figure:**
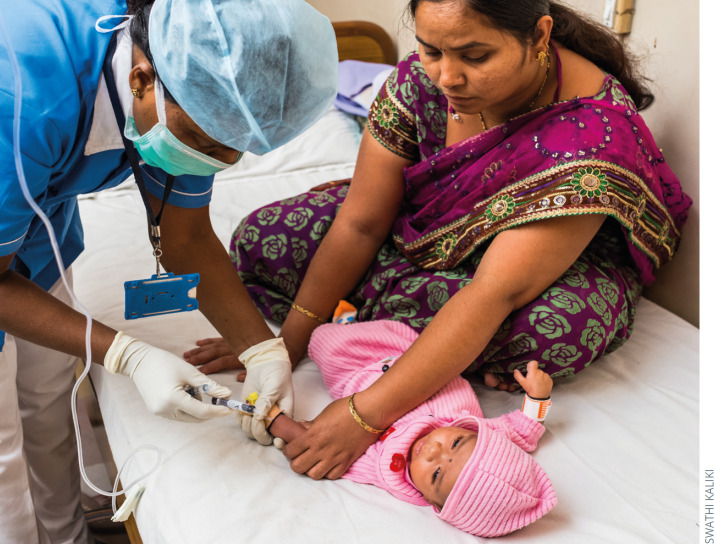
A baby is prepared for chemotherapy. INDIA

We hope that you will also discuss the questions with your colleagues and other members of the eye care team, perhaps in a journal club. To complete the activities online - and get instant feedback - please visit **www.cehjournal.org**

**Tick ALL that are TRUE**

**Question 1 The following are clinical features of retinoblastoma in a child aged 18 months:**
  □ **a.** white-yellow pupil reflex
  □ **b.** strabismus
  □ **c.** painful red eye
  □ **d.** decreased visual acuity
  □ **e.** proptosis
**Question 2 The following are true for germline (hereditary) retinoblastoma:**
  □ **a.** there is always a history of another family member having retinoblastoma
  □ **b.** tumours are often in both eyes
  □ **c.** there is a risk of other types of cancer in later life
  □ **d.** tumours are often multi-focal
  □ **e.** children present at an older age than non-hereditary cases.
**Question 3 The following are methods of treating intra-ocular (stage 0 or 1) retinoblastoma:**
  □ **a.** external beam radiotherapy
  □ **b.** intra-vitreal chemotherapy
  □ **c.** enucleation
  □ **d.** laser photocoagulation
  □ **e.** systemic chemotherapy.
**Question 4 The following are methods of treating extra-ocular (stage 2, 3, 4) retinoblastoma:**
  □ **a.** systemic chemotherapy
  □ **b.** intra-vitreal chemotherapy
  □ **c.** external beam radiotherapy
  □ **d.** laser photocoagulation
  □ **e.** exenteration.

## ANSWERS

1. a, b, c and e are TRUE. A child of 18 months will not present with decreased visual acuity. A child with Rb may present with a turned in or turned out eye (strabismus), a white, yellow reflex in the pupil (leukocoria), a red painful eye due to secondary glaucoma from intra-ocular tumour or a protrusion of the eye due to spread of the tumour into the orbit.
2. b, c and d are TRUE. Germline (hereditary) disease may present as a first case with no previous family history. In germline retinoblastoma the children are usually younger at presentation (median age of 15 months as compared to 24 months for non-germline cases). Bilateral, multifocal disease is common in germline (hereditary) disease. As all cells in the body already have one Rb gene mutation, second cancers in other sites may occur later in life.
3. b, c, d and e are TRUE. External beam radiotherapy is no longer used to treat Rb confined to the globe, as it was found to be associated with an increased risk of cancers in patients with germline retinoblastoma.
4. a, c, and e are TRUE. If the disease has spread beyond the globe then intravitreal chemotherapy or laser photocoagulation are not appropriate.

